# Neural activation during emotional interference corresponds to emotion dysregulation in stressed teachers

**DOI:** 10.1038/s41539-022-00123-0

**Published:** 2022-04-20

**Authors:** Samuel Fynes-Clinton, Chase Sherwell, Maryam Ziaei, Ashley York, Emma Sanders O’Connor, Kylee Forrest, Libby Flynn, Julie Bower, David Reutens, Annemaree Carroll

**Affiliations:** 1 Centre for Advanced Imaging, The University of Queensland, Brisbane, QLD Australia; 2 School of Education, The University of Queensland, Brisbane, QLD Australia; 3 School of Psychology, The University of Queensland, Brisbane, QLD Australia

**Keywords:** Education, Emotion

## Abstract

Teacher stress and burnout has been associated with low job satisfaction, reduced emotional wellbeing, and poor student learning outcomes. Prolonged stress is associated with emotion dysregulation and has thus become a focus of stress interventions. This study examines emotional interference effects in a group of teachers suffering from high stress and to explore how individual differences in cognitive control, emotion dysregulation, and emotion recognition related to patterns of neural activation. Forty-nine teachers suffering moderate-high stress participated in an emotional counting Stroop task while their brain activity was imaged using functional magnetic resonance imaging. Participants viewed general or teacher specific words of either negative or neutral valence and were required to count the number of words on screen. Behavioural and neuroimaging results suggest that teachers *are* able to control emotional responses to negative stimuli, as no evidence of emotional interference was detected. However, patterns of neural activation revealed early shared engagement of regions involved in cognitive reappraisal during negative task conditions and unique late engagement of the hippocampus only while counting teacher-specific negative words. Further, we identified that greater emotion dysregulation was associated with increased activation of regions involved in cognitive control processes during neutral word trials. Teachers who showed slower emotion recognition performance were also found to have greater activation in regions associated with visual and word processing, specifically during the teacher specific negative word condition of the task. Future research should explore emotion regulation strategy use in teachers and utilise temporally sensitive neuroimaging techniques to further understand these findings.

## Introduction

Teaching has been identified as one of the most stressful professions^1–3^, and teacher stress and burnout has become recognised as a major contributor to rates of teacher attrition^4–6^. The retention of qualified educators is a global concern^7^ that has been investigated through many diverse theoretical approaches^8^. Sources of teacher stress are varied, with commonly cited causes being high workload and time pressure^9,10^, the highly interpersonal nature of the job^11–13^, role ambiguity and conflicts^14^, and the large emotional demands placed on teachers^15^. As well as contributing to burnout and attrition, teacher stress has been associated with reduced teacher self-efficacy^16,17^ and student learning outcomes^18^. While external factors play a large role in teacher stress, individual resilience to stress and burnout is an important predictor of teacher wellbeing^19,20^. Although reducing sources of teacher stress is important, training teachers to develop and employ strategies aimed at building resilience could have a more broad and sustained impact on overall job satisfaction and emotional wellbeing. Interventions designed to address teacher stress vary greatly in approach considering the multivariate nature of teacher stress^21^. However, building socio-emotional strategies for managing negative or unwanted emotions has gained recent attention^22^. To fully assess the utility of such approaches, it is critical to understand the role that executive functions governing affective regulation and cognitive control play in teacher stress.

The ability to control or regulate one’s emotions has been identified as a protective factor against teacher stress^23–25^. Emotion regulation refers to the ability to up- or down-regulate one’s own or another’s emotional responses, either in duration or magnitude through behavioural or cognitive control^26^. More broadly, Gratz and Roemer^27^ conceptualise emotion regulation as an array of skills and processes that allow a) awareness and understanding of emotions, b) acceptance of emotions, c) the ability to perform goal-directed behaviour in the presence of negative emotions, and d) flexible use of emotional regulation strategies to modulate emotional responses. Emotion dysregulation refers to deficits or impairments in any of these skills, with a focus on the inability to regulate negative emotions. Difficulties in managing negative or challenging emotions, is a predictor of burnout in emotionally challenging professions, including teaching^28–30^.

The prominent theoretical perspective on emotional intelligence posits that emotion recognition causally precedes emotional understanding and, in turn, emotion regulation^31,32^. Although related, self-reported emotion recognition and emotion regulation abilities have been found to independently predict outcomes for adjusting to novel situations^33^. Understanding the relationship between different aspects of emotional intelligence and neural activity during emotion interference could have important implications for the development of targeted stress management and preventative strategies.

The information-processing model of emotion regulation posits that each stage within the affective appraisal of a situation (i.e., attention, appraisal, response) represents a potential target for emotion regulation strategies^34^. The initial stage of information processing can be modified through *attentional selection*, whereby attention can be directed to or disengaged from emotional elements of a situation^35–37^. Selectively disengaging from affective stimuli is an early and conscious regulatory measure that can prevent further emotion generation. Conversely, the appraisal of emotion-generating stimuli is the target of *reappraisal* strategies, where an individual reflects on a situation in order to alter their initial emotional response. Recent evidence indicates that attentional selection and reappraisal strategies both decrease the intensity of negative emotions but do not differ in efficiency^38^.

In pedagogical practice, such cognitive strategies are often referred to as *deep acting* strategies of emotional labour, and are associated with decreased burnout symptoms in teachers^39^. In contrast, the use of *surface acting* strategies that involve the suppression or faking of emotions are positively associated with teacher burnout^15^. The majority of teachers feel they do not receive adequate support in developing effective strategies for managing their own emotions^40^. The consequence likely being the active suppression of emotional distress that has been shown to result in occupational burnout^20^.

Beyond emotional dysregulation, occupational stress and burnout affects a broader range of self-regulatory and cognitive capabilities. According to Job Demand-Resource theory, attentional and cognitive deficits result from stress and burnout due to the compensatory cost of mental and physical exhaustion from working in a high demand environment/organisation^41^. More recently it was proposed that job strain is related to greater engagement in maladaptive self-regulatory behaviours, and less adaptive self-regulation thus exacerbating burnout^42^. Bakker and de Vries^42^ suggest that the ability to recognise and regulate emotions (i.e., emotional intelligence), and the ability to proactively effect change in the environment (i.e., proactive personality) can moderate the link between job strain and maladaptive emotion regulation practices. Evidence from self-report and psychometric tests indicate significant deficits in executive functioning, attention, and memory^43,44^. Experimental evidence of the effects of burnout on neural processes related to cognitive control show increased cognitive load^45^, attentional deficits^46^, and increased processing of errors and negative feedback^47^.

Cognitive control and attentional capabilities play an important role in effective emotion regulation strategies. Emotional stimuli capture attention automatically, and greater cognitive effort is required to redirect attention in emotion eliciting situations^48^. Emotional information biases the competition for information processing resources^49^, whereby interference from emotionally salient stimuli creates cognitive conflict compromising our ability to process task relevant information and complete tasks that require cognitive control^50,51^. Critically, cognitive control has been shown to be deficient in individuals experiencing occupational burnout^43,44^. Teachers experiencing burnout also display attentional deficits in cognitive tasks, including difficulties with inhibition and increased distractibility^52^. Considering the importance of attentional selection as a means of emotion regulation, poorer cognitive control in stressed teachers may act as a precursor to or exacerbate emotional dysregulation.

The automatic attentional capture by emotional information is frequently investigated using the emotional Stroop task^53^. An adaptation of the ‘classic’ Stroop paradigm, participants are tasked with identifying one particular aspect (e.g., the number, or colour) of a visual stimulus, typically words. Rather than cognitive interference occurring due to conflict between strongly automatic information processes (i.e., naming the colour of a word rather than reading the word), participants are slower at identifying the task-relevant information due to distraction from emotion-eliciting information within the stimulus (emotion eliciting words). Slower reaction times when identifying features of emotionally laden words compared to neutral words is typically regarded as a measure of emotional interference due to the automatic attentional capture^50^. In clinical populations, the presentation of disorder specific stimuli (i.e., words relevant to obsessive compulsive disorder or post-traumatic stress disorder) has been shown to enhance emotional interference effects (see review^54^). Resolving the cognitive conflict due to emotional distraction relies on a shared neural network, as shown by functional magnetic resonance imaging (fMRI) studies using emotional Stroop paradigms^55–58^. Meta-analyses of such studies have shown consistent engagement of networks within the prefrontal cortex^51,59^. Specifically, the ventrolateral, dorsomedial, and dorsolateral regions associated with conflict monitoring and resolution showed robust engagement across studies during emotional interference.

While there is a broad literature associating occupational burnout with reduced emotional and cognitive functioning, investigations into the underlying neural and cognitive processes that result from or contribute to teacher burnout are scarce. It is critical for our understanding of the contributing factors to teacher stress, as well as the development of effective preventative measures, to examine how teachers experiencing burnout process emotional information. Specifically, we aimed to characterise the neural correlates of emotion interference in teachers experiencing moderate to high stress. For this purpose, practising teachers who reported high levels of stress and professional burnout completed an emotional counting Stroop task (adapted from the paradigm by Whalen and colleagues)^60^ while undergoing fMRI. The ability to disengage from emotion-eliciting events and reappraise emotions, represent potential markers of emotional regulation that may be critical in protecting against stress and burnout. Thus, a second goal of this study was to examine how individual differences in self-reported emotion dysregulation relate to patterns of neural activity during an emotion interference task. Finally, we explored the relationship between neural activation patterns on the emotion interference task and performance on (1) an emotion recognition task, and (2) a cognitive control task. Emotion regulation encapsulates a range of abilities, including cognitive control and emotion recognition; disambiguating the role of these abilities in emotion interference could inform the development of strategies for the management of teacher stress and burnout.

Using multivariate Partial Least Squares (PLS; https://www.rotman-baycrest.on.ca/index.php?section=84) analysis, we identified spatiotemporal patterns of brain activation functionally associated with emotional interference in our cohort of teachers experiencing significant stress and burnout (as confirmed by scores on the Perceived Stress Scale). Participants were asked to respond to stimuli by pressing a button to indicate the number of times each word was presented on the screen whilst disregarding word meaning. Participants viewed general negative words (e.g., torture) and neutral words (e.g., book) as well as teacher specific negative words (e.g., marking) and neutral words (e.g., student). The control condition involved viewing numbers in their written form which were congruent with the number of times the word was presented on screen (e.g., three, three, three). Like clinical studies using the emotional Stroop task, teacher-specific words were included as stimuli for the purpose of delineating differences between occupationally specific emotional interference and emotional interference resulting from valence more generally. Emotion eliciting stimuli should draw attention from the unrelated primary task of counting the words, leading to emotional interference^50^, resulting in comparatively slower reaction times. Based on prior research exploring emotional interference, we hypothesised that interference from teacher specific negative, and (to a lesser degree) general negative words would be related to increased activation of the amygdala, fusiform gyrus, dorsolateral prefrontal cortex (DLPFC), insular cortex, anterior cingulate cortex (ACC), and medial frontal gyrus^51^.

Due to the automatic capture of attention associated with negative words, we predicted that faster attention switching performance (a measure of cognitive control) would correspond to greater prefrontal engagement during interference trials. Further, we expected this effect would be stronger for teacher-specific words compared to general words. We also anticipated that people with faster emotion recognition performance would show reduced reliance on visual and emotion processing regions during negative task conditions, reflecting reduced demand with greater capability. Considering the importance of attentional selection as a means of emotion regulation, we anticipated that greater emotion dysregulation (as assessed by the Difficulties in Emotion Regulation Scale, or DERS)^27^ would be associated with diminished ability to disengage from emotional stimuli, as demonstrated by emotional interference in the emotional Stroop task. Specifically, we predicted that greater self-reported emotion dysregulation would correlate with increased activity in the amygdala^61^ and decreased activity in the DLPFC when viewing negative compared to neutral words^38^. If the ability to disengage from negative emotional stimuli plays a critical role in mitigating stress through emotion regulation, we expected that teachers displaying emotion dysregulation would display greater engagement of brain regions related to attentional capture due to emotional interference (for example, the amygdala and rostral ACC).

## Results

### Correlations with emotional counting Stroop task performance

Participants’ ages ranged from 25 to 67 years of age and on average, teachers had 18 years of teaching experience. After controlling for age-related differences in reaction time we sought to explore whether years of teaching experience related to stress, emotion regulation and/or response latencies on the emotion interference task (Table 1). Bivariate (Pearson’s r) correlation analysis revealed a strong positive correlation between age and years of teaching experience (*r* = 0.82, *p* < 0.001). Controlling for the effect of age, partial correlation analysis revealed that years of teaching experience and participants’ median overall reaction time on the Emotion Recognition Task (ERT) were positively correlated (*r* = 0.31*, p* < 0.05). Greater years of teaching experience corresponded to slower reaction times in emotional Stroop task and therefore poorer emotion recognition. A significant positive correlation was found between reported stress as measured on the Perceived Stress Scale (PSS^62^) and emotion dysregulation as measured by the total score on the DERS^27^, indicating that teachers who reported greater stress, displayed greater emotion dysregulation. Reaction time on the emotional Stroop task was intercorrelated among task conditions but did not correlate significantly with any other behavioural or demographic variable.

**Table 1:** Pearson’s correlations after controlling for the influence of age, between response latencies on the emotional counting Stroop task, stress, emotion regulation, and years of teaching.

Measures	1	2	3	4	5	6	7	8	9
1. Years Teaching	–	−0.11	0.07	0.31^b^	0.19	−0.10	−0.04	−0.16	−0.18
2. Stress (PSS)		–	0.46^a^	−0.16	0.26	−0.12	−0.08	0.06	−0.13
3. Emotion Regulation (DERS Total)			–	0.10	0.11	−0.02	0.11	0.18	0.09
4. Emotion Recognition RT (ERT)				–	0.12	−0.10	−0.08	−0.01	−0.12
5. Cognitive Control RT (AST)					–	−0.18	0.09	0.09	−0.02
6. General Negative RT (Task)^c^						–	0.58^a^	0.62^a^	0.72^a^
7. General Neutral RT (Task)^c^							–	0.67^a^	0.63^a^
8. Specific Negative RT (Task)^c^								–	0.71^a^
9. Specific Neutral RT (Task)^c^									–

*PSS* Perceived Stress Scale score, *DERS* Difficulties in Emotion Regulation Scale score, *ERT* Emotion Recognition Task, *AST* attention switching task, RT reaction time.

^a^Correlation is significant at the 0.01 level (2-tailed).

^b^Correlation is significant at the 0.05 level (2-tailed).

^c^Reaction time relative to control task.

### Emotional counting Stroop task performance

Mean reaction times and accuracy scores for each condition of the emotional Stroop task were calculated relative to the control condition of the task (i.e., reaction time on general-negative words minus control condition). The control condition used the number of items as the word stimuli (the word ‘three’ repeated three times) as a baseline measure of reaction time. Reaction time and accuracy, normalised to the control, were both analysed using a one-way repeated measures analysis of variance (RM-ANOVA) where the within-subject factor, *condition*, had four levels: General negative words (GenNeg); General neutral words (GenNeut); Teacher-specific negative words (SpeNeg); and Teacher-specific neutral words (SpeNeut). The analyses of reaction times revealed no significant main effect of task condition, *F(3,144) =* 0.820*, p* > *0.05, ns*. The analysis of accuracy revealed a significant main effect of condition, *F(3, 144)* *=* 37.24*, p* < *0.001*, η_p_^2^ = 0.44. Follow-up paired *t*-tests, Bonferroni corrected for multiple comparisons, revealed that accuracy in the SpeNeut condition relative to the control condition (*M* = *−3.71; SD 2.95)* was significantly lower than that observed in the GenNeg (*M* = *−0.20; SD* = *2.92)* condition (t(48) = 9.37, *p* < *0.001)*, GenNeut (*M* = *−0.58; SD* = *2*.78) condition (t(48) = 7.17, *p* < *0.001)*, and SpeNeg (*M* = *−0.10; SD* = *2.19)* condition (t(48) = 9.63, *p* < *0.001)*. In all conditions except SpeNeut, the percentage of correct responses was greater than 97%, indicating a ceiling effect.

### Task-related functional activation

#### All task conditions

Functional brain images were analysed using whole-brain partial least squares (PLS) analyses, which decomposes all data across participants into an orthogonal set of latent variables (LVs) using singular value decomposition (SVD). This data-driven approach does not rely on the specification of contrasts between conditions, but rather differentiates patterns of neural activity based on the maximal covariance associated with experimental tasks and covariates. Similar to principle components analysis, the initial LV captures the largest amount of covariance present in the dataset, followed by diminishing proportions in subsequent variables. The resulting LVs provide a spatiotemporal pattern of voxel saliences, or brain patterns, depicting the voxels associated with the experimental task or other covariates. Calculated set weightings indicate how strongly such patterns corresponds to experimental conditions or covariates, and the degree of covariance in neural activity explained by the LV. Brain scores are derived that reflect the extent to which each participant expresses the pattern represented by the LV (see *Method* for full details).

The first analysis, including all task conditions and the control condition, revealed one LV accounting for 36.32% of covariance in the data that was significant by permutation (*p* = 0.014). This LV differentiated neural activity engaged during the SpeNeut condition from a pattern of activation engaged during the SpeNeg condition of the task (Supplementary Fig. 1B). Specifically, significant differentiation was observed 3–6 s after stimulus onset. As seen in Supplementary Fig. 1A and Supplementary Table 1, SpeNeut word counting was associated with greater activation in the right frontal pole, middle frontal gyrus, inferior temporal gyrus, temporal pole, supramarginal gyrus, subcallosal cortex, and lateral occipital cortex. Left hemispheric peak activations were localised in the hippocampus, amygdala, and brain stem, while bilateral activation was observed in the middle temporal gyrus, precuneus, cingulate gyrus, thalamus, and putamen. Activation of this network was anti-correlated with activation of the body of the right caudate nucleus engaged uniquely during the SpeNeg word counting condition.

PLS defines a set of mutually orthogonal LVs meaning that no two LVs can correlate with one another and variance assigned to one LV will not be included in the results of subsequent LVs. Because the first analysis accounted for so much of the variance associated with the SpeNeg condition, effects relating to the differences between general and specific word types, may not have been detected. With the aim of assessing the relationship between the GenNeg and SpeNeg task conditions, a second PLS analysis was conducted, excluding the SpeNeut condition. By excluding the SpeNeut condition variance associated with the SpeNeg condition was redistributed.

**Fig. 1:**
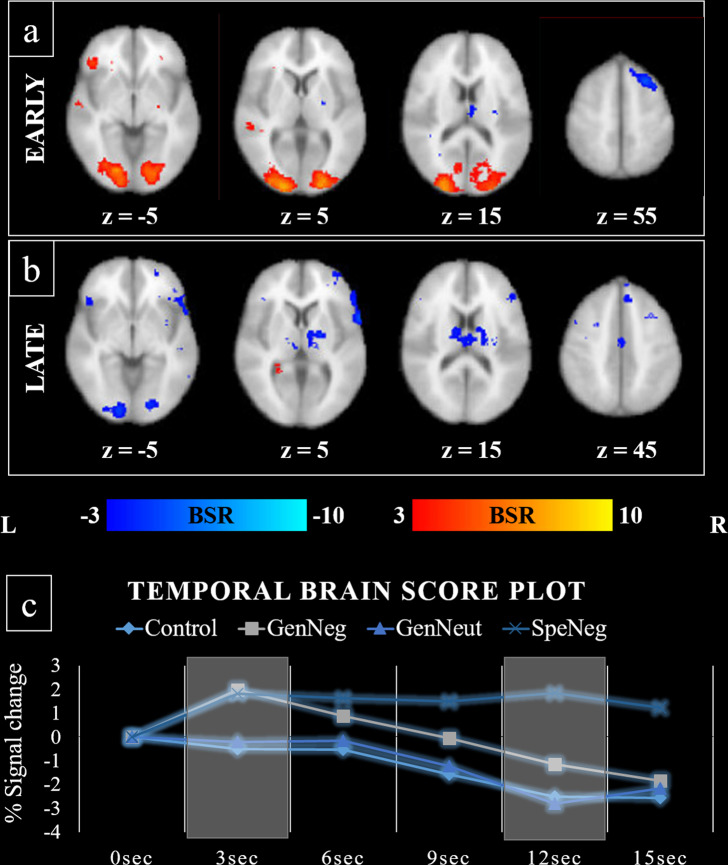
Neural correlates of emotional interference. Whole-brain spatiotemporal PLS results including the control, general negative, general neutral, and specific negative emotional Stroop task conditions. **a**, **b** Latent variable voxel salience overlaid on the 2 mm MNI-average brain template. **a** Early peak activation occurred 3–6 s post stimulus onset whereas a (**b**) late peak in activation occurred 12–15 s post stimulus onset. BSR bootstrap ratio tests of significance, L left hemisphere, R right hemisphere, z axial slice number. **c** The temporal brain score plot reflecting percentage signal change from 0, starting from the stimulus onset and progressing in 3 s time increments. Values greater than 0 correspond to positive voxel saliences in **a** and **b** (red) and values less than 0 correspond to negative voxel saliences in **a** and **b** (blue).

This second analysis yielded a significant LV that accounted for 42.06% of covariance in the data (*p* = 0.032). Two peaks of activation were observed in the spatiotemporal pattern of activation (Fig. 1C). The first peak occurred 3–6 s after stimulus onset and corresponded to a common pattern of activation for general and specific negative words. Early activation during negative word counting was associated with greater activation in the left orbitofrontal cortex (OFC), middle and superior temporal gyri, precuneus, and cuneus. Two large clusters of activation were also detected in the bilateral occipital poles (peak) which extended bilaterally to the superior and inferior portions of the lateral occipital cortices, lingual gyrus and fusiform gyrus. Negative word counting was also associated with reduced activation in the bilateral thalamus and right; superior frontal gyrus (extending to middle frontal gyrus), precentral gyrus, cingulate gyrus, and angular gyrus (Fig. 1A, Table 2A).

**Table 2:** Peak activations and coordinates reflecting condition differences in the spatiotemporal pattern of neural activation during emotional interference.

A. Control, GenNeg, GenNeut, SpeNeg: (Lag 1: 3–6 s)							
		MNI coordinates					
Region	Hem	*x*	*y*	*z*	BSR	Cluster size	*p* val
**Specific Negative (Positive Saliences)**							
OFC	L	−42	32	−2	4.98	178	<0.001
MTG (anterior division)	L	−64	0	−14	4.15	24	<0.001
STG (posterior division)	L	−56	−32	4	3.98	31	<0.001
Precuneus	L	−6	−54	28	4.03	47	<0.001
Cuneus	L	−12	−74	16	4.2	71	<0.001
Occipital pole	L	−22	−96	8	8.51	2289	<0.001
	R	22	−96	4	7.52	2138	<0.001
**Control & General Neutral (Negative Saliences)**							
SFG	R	24	20	54	−5.41	381	<0.001
Precentral Gyrus	R	16	−18	76	−4.6	29	<0.001
Lateral Occipital Cortex (superior division)	R	44	−62	36	−3.91	34	<0.001
CG (anterior division)	R	8	−6	32	−4.09	32	<0.001
Thalamus	L	−12	−10	20	−3.38	22	<0.001
	R	6	−10	16	−3.7	35	<0.001

B. Control, GenNeg, GenNeut, SpeNeg: (Lag 4: 12–15 s)							
		MNI coordinates					
Region	Hem	x	y	z	BSR	Cluster size	*p* val
**Specific Negative (Positive Saliences)**							
Hippocampus	L	−26	−40	2	3.85	21	<0.001
**Control & General Neutral (Negative Saliences)**							
Frontal Pole	R	4	46	50	−4.97	248	<0.001
OFC	L	−48	26	−8	−3.58	36	<0.001
	R	32	28	−10	−4.1	102	<0.001
IFG (pars triangularis)	L	−50	26	20	−3.73	94	<0.001
IFG (pars opercularis)	R	62	12	4	−4.98	593	<0.001
SFG	L	−10	−2	76	−5.34	319	<0.001
	R	16	14	68	−3.79	34	<0.001
Precentral Gyrus	L	−52	10	36	−3.35	39	<0.001
Postcentral Gyrus	L	−8	−36	76	−4.59	66	<0.001
	R	20	−44	74	−3.4	20	<0.001
ITG (temporooccipital part)	R	48	−52	−16	−4.95	282	<0.001
MTG (posterior division)	R	48	−22	−16	−4.07	95	<0.001
IC	R	36	0	−12	−4.66	118	<0.001
Temporal Occipital Fusiform Cortex	L	−44	−48	−22	−3.81	27	<0.001
Occipital Fusiform Gyrus	L	−32	−72	−14	−3.75	20	<0.001
	R	18	−86	−6	−4.53	164	<0.001
Occipital pole	L	−14	−92	−8	−5.12	312	<0.001
CG (anterior division)	L	−2	−10	40	−3.97	114	<0.001
	R	2	12	24	−4.04	128	<0.001
CG (posterior division)	R	4	−32	26	−4.08	54	<0.001
Pallidum	R	20	−6	4	−4.58	712	<0.001
Cerebellum	L	−40	−70	−30	−3.78	50	<0.001
	R	22	−54	−32	−4.18	33	<0.001

Results for **A** taken from lag one of the spatiotemporal analysis corresponding to 3–6 s after stimulus onset. Results for **B** taken from lag one of the spatiotemporal analysis corresponding to 12–15 s after stimulus onset. Nomenclature correspond to the Harvard-Oxford cortical and subcortical structural atlases.

*OFC* orbitofrontal cortex, *ITG* inferior temporal gyrus, *MTG* middle temporal gyrus, *STG* superior temporal gyrus, *IFG* inferior frontal gyrus, *SFG* superior frontal gyrus, *CG* cingulate gyrus, *IC* insular cortex, *BSR* bootstrap ratio from the PLS analysis, *Hem* hemisphere, *R* right, *L* left, *p val* estimated p value from the BSR.

The second later peak occurred 12–15 s after the stimulus onset differentiating the SpeNeg condition from activation associated with the control, GenNeut, and GenNeg conditions of the task. The late peak was associated with activation in the hippocampus for the SpeNeg condition only, which differentiated from a pattern of increased engagement during general negative, general neutral and the control condition. Activations for non-interference (neutral word) trials involved the bilateral OFC, inferior frontal cortex (pars triangularis and pars opercularis), superior frontal gyrus, postcentral gyrus, fusiform gyrus (occipital portion), cingulate gyrus, and cerebellum. Activations were also observed in the right frontal pole (extending to the superior frontal gyrus), right inferior and middle temporal gyri, right insular cortex, right pallidum (including thalamus), left precentral gyrus, left fusiform gyrus (temporal part), and left occipital pole (extending to lingual gyrus and fusiform gyrus) (Fig. 1B, Table 2B).

### Brain-behaviour analysis of individual differences

Three separate brain-behaviour PLS analyses were conducted to explore the relationship between emotional interference and individual differences in emotion dysregulation. These analyses explored the correlation between neural activation and (1) total scores on the DERS a measure of participants self-reported difficulties in emotion regulation, (2) response latency on the ERT which assesses emotion labelling speed, and (3) response latency on a measure of cognitive control, the Attention Switching Task (AST). As our task-related PLS analyses revealed that the inclusion of the SpeNeut condition obscured spatiotemporal patterns of activation associated with the interference effect of interest, it was not included in further analyses.

#### Emotion dysregulation

**Fig. 2:**
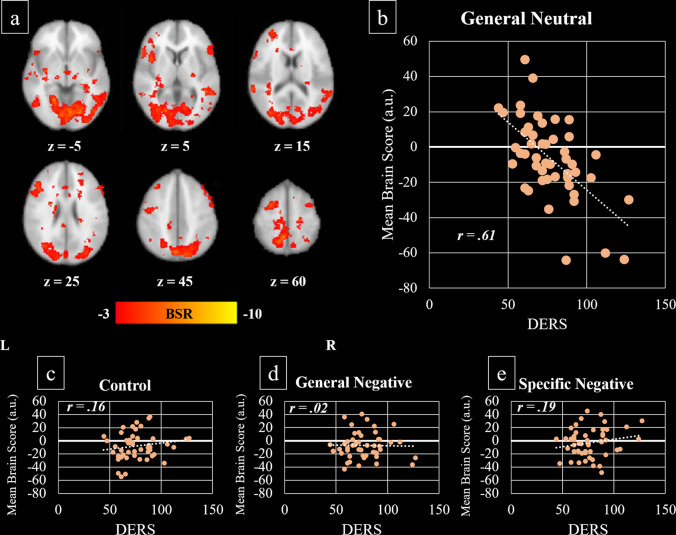
Covariance of brain activity with DERS during the emotional Stroop task. **a** Latent variable voxel salience overlaid on the 2 mm MNI-average brain template. BSR bootstrap ratio tests of significance, L left hemisphere, R right hemisphere, z axial slice number. **b**–**e** Negative correlation between mean brain scores (a.u. = arbitrary units) and scores on the DERS. People with more difficulties in emotion regulation (higher DERS scores) showed greater engagement of the activation pattern (**a**) during the general neutral condition of the task (**b**) and reduced engagement of these regions during the specific negative condition (**e**).

The brain-behaviour PLS analysis exploring the relationship between emotion dysregulation (total score on the DERS) and functional activation related to the emotional Stroop task (Control, GenNeg, GenNeut, and SpeNeg conditions) revealed one significant LV (*p* < *0.001*) accounting for 51.38% of covariance in the data. As shown in Fig. 2A, corresponding brain activations occurring 9–12 s after stimulus onset included a large cluster of activation comprising bilateral lingual gyrus, occipital fusiform gyrus, precuneus, cuneus, lateral occipital cortices and occipital poles. Activation was also identified in the bilateral angular gyrus, frontal pole, superior and middle frontal gyri, the right pars opecularis and pars triangularis of the inferior frontal gyrus, the right superior temporal gyrus, left middle temporal gyrus, left supramarginal gyrus, left central opercular cortex/insular cortex, thalamus and cerebellum (Table 3). Pearson’s correlation revealed that during the GenNeut condition, greater activation in the aforementioned regions was associated with higher DERS (reflecting greater emotion dysregulation) whereas low DERS scores related to reduced activation in these regions, *r* = −0.61 (Fig. 2B). In contrast, a marginal reversal of this effect was observed during the SpeNeg (*r* = 0.19) and control (*r* = 0.16) conditions of the emotional counting Stroop task suggesting that lower emotion dysregulation is related to increased activity across the aforementioned network of brain regions during these conditions.

**Table 3:** Regions engaged during the emotional interference task that are significantly associated with lower self-reported difficulties in emotion regulation.

Significant correlations with DERS: (Lag 3: 9–12 s)							
		MNI coordinates					
Region	Hem	*x*	*y*	*z*	BSR	Cluster size	*p* val
LG	R	14	−68	−12	−8.82	12170	<0.001
Precuneus	L	−10	−54	62	−7.16	2333	<0.001
Frontal Pole	L	−32	52	8	−4.99	381	<0.001
	R	30	40	40	−3.71	21	<0.001
SFG	L	−22	0	68	−6.71	461	<0.001
	R	18	0	66	−4.53	36	<0.001
MFG	L	−34	32	46	−6.56	526	<0.001
	R	44	10	54	−7.09	660	<0.001
IFG, pars opercularis	R	54	20	4	−4.6	162	<0.001
Precentral Gyrus	L	−50	−10	52	−6.67	598	<0.001
	R	20	−22	60	−5.54	249	<0.001
STG, posterior division	R	62	−14	−2	−4.61	45	<0.001
MTG, posterior division	L	−66	−24	−4	−4.9	36	<0.001
MTG, temporooccipital part	L	−56	−48	2	−3.8	41	<0.001
ITG, anterior division	R	50	−12	−24	−3.9	21	<0.001
Central Opercular Cortex	L	−44	10	4	−5.82	362	<0.001
AG	L	−46	−46	56	−4.43	34	<0.001
	R	46	−52	44	−3.61	21	<0.001
Supramarginal Gyrus, posterior division	L	−38	−42	16	−6.19	700	<0.001
Paracingulate Gyrus	L	−8	28	32	−4.46	118	<0.001
Thalamus	R	2	−12	−8	−4.17	38	<0.001
Pallidum	R	20	−16	0	−6.11	119	<0.001
Putamen	L	−30	−24	0	−6.64	581	<0.001
Cerebellum	R	14	−38	−26	−4.37	24	<0.001

All results were taken from lag three of the spatiotemporal analysis corresponding to 9–12 s after stimulus onset. Nomenclature correspond to the Harvard-Oxford cortical and subcortical structural atlases.

*LG* lingual gyrus, *SFG* superior frontal gyrus, *MFG* middle frontal gyrus, *IFG* inferior frontal gyrus, *STG* superior temporal gyrus, *MTG* middle temporal gyrus, *ITG* inferior temporal gyrus, *AG* angular gyrus, *BSR* bootstrap ratio from the PLS analysis, *Hem* hemisphere, *R* right, *L* left, *p val* estimated *p* value from the BSR.

#### Emotion recognition

**Fig. 3:**
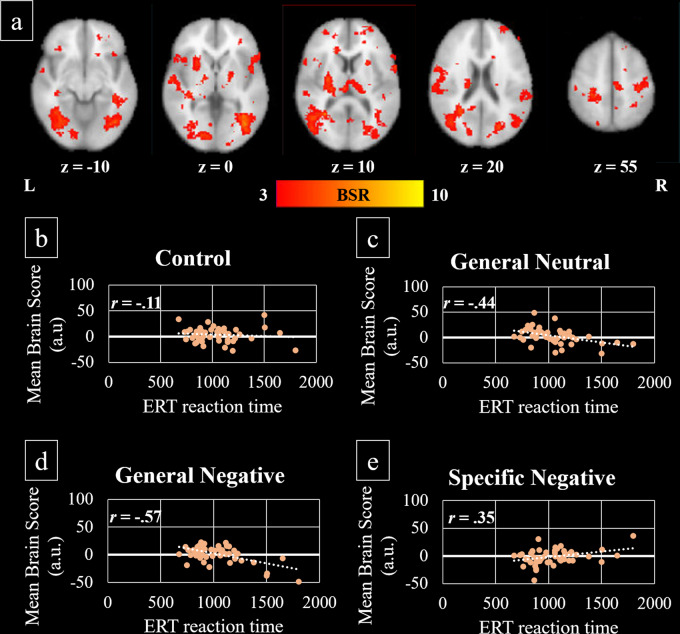
Covariance of brain activity with reaction time performance on the ERT during the emotional Stroop task. **a** Latent variable voxel salience overlaid on the 2 mm MNI-average brain template. BSR bootstrap ratio tests of significance, L left hemisphere, R right hemisphere, z axial slice number. **b**–**e** Correlation between mean brain scores (a.u. = arbitrary units) and reaction time performance on the ERT. Faster reaction time on the ERT related to higher mean brain scores during the general neutral (**c**) and general negative condition (**d**) but lower mean brain scores during the specific negative condition (**e**). Higher mean brain scores correspond to greater engagement of regions (red) in **a**.

The brain-behaviour PLS analysis including the control, GenNeg, GenNeut, and SpeNeg conditions of the emotional Stroop task and median reaction time on the ERT revealed one significant LV (*p* = 0.012). This LV accounted for 35.72% of covariance in the data and revealed a broad pattern of activation 12–15 s after stimulus onset. As shown in Fig. 3A, this network included right lateral occipital cortex, left precuneus (unilaterally extending to angular gyrus and lateral occipital cortex), bilateral supramarginal gyri, right superior parietal lobule, bilateral middle temporal gyrus, left superior temporal gyrus, bilateral postcentral and precentral gyri, bilateral superior frontal gyrus, right middle frontal gyrus and pars triangularis, bilateral OFC, right caudate (body and head), bilateral cingulate (anterior and posterior) and paracingulate gyri, thalamus and cerebellum (Supplementary Table 2). Activation of this network appeared to be differentially dependent on an individual’s emotion recognition according to the task stimuli (Fig. 3B–E). Faster reaction times on the ERT were strongly correlated with increased activation in these regions during GenNeg trials (*r* = −0.57) and moderately so during GenNeut trials (*r* = −0.44). Conversely, a moderate correlation indicated that slower emotion recognition performance was associated with increased activation of this network during SpeNeg trials (*r* = 0.35).

#### Cognitive control

Mean reaction times on the AST were calculated as a measure of cognitive control. The brain-behaviour PLS analysis of covariance between brain activity during the emotional Stroop task and cognitive control revealed no significant LVs.

## Discussion

Strong emotion regulation, such as the ability to identify and control one’s responses to emotions, has been recognised as a protective factor against stress and burnout in teachers^63,64^. On the other hand, emotion dysregulation is associated with maladaptive social and interpersonal emotional responses, resulting from heightened sensitivity, greater emotional reactivity, and an inability to down-regulate the emotional response^65^. In teachers, chronic stress has also been associated with a reduced capacity for emotion regulation^12^. Being able to recognise emotions and redeploy attention during the initial stages of information processing is one strategy for emotion regulation^22,35,37,38^. The primary aim of this study was to explore the neural correlates of emotional interference in a group of teachers reporting moderate-high perceived stress. Specifically, we aimed to characterise patterns of neural activation during general and job specific emotion interference. We also examine the way in which individual differences in emotion dysregulation, emotion recognition, and cognitive control are related to neural activation during an emotional counting Stroop task. While we did not observe behavioural interference effects in the emotional Stroop task, whole-brain task analysis revealed that teachers engaged regions involved in cognitive control. The behaviour-PLS analyses show differential activation of brain regions between task conditions that provide meaningful insight into how individuals experiencing stress and burnout process emotionally laden stimuli.

According to Job Demand-Resource theory, burnout occurs as a result of depleting or inadequate resources and high psychological, physical, and emotional job demands^41^. In this study, correlations between behavioural variables suggest that greater experience in a teaching profession may reduce the ability to recognise emotional faces and across all experience levels, greater stress is associated with increased emotion dysregulation. One implication of this study is that time spent in a high demand work-role such as teaching may increase depersonalisation. Depersonalisation is also linked to more stressful employee-client interactions^66^. In a recent study it was revealed that reductions in stress and burnout of teachers was associated with greater academic self-perception in students and increased feelings of teacher support in the classroom^67^. In a meta-analysis exploring the effectiveness of burnout interventions it was found that individual cognitive/behavioural interventions produced small but reliable effects on general employee burnout and exhaustion, but not depersonalisation or professional efficacy^68^. An important avenue for future research is to examine whether interventions targeting organisation-level job resources^42^ impact levels of depersonalisation among teachers.

Given that the participants of this study were a non-clinical sample, it could be expected that any behavioural interference effect of the emotional Stroop task would be small, if at all detectable. In line with this, we did not observe any behavioural interference effects on the emotional Stroop task and identified a clear ceiling effect in all conditions except the specific neutral condition where accuracy was marginally reduced. Emotional Stroop tasks do not induce cognitive interference in the same manner as traditional Stroop tasks. Emotional word stimuli are not semantically related to the task-relevant information (number of words) nor do they interfere with response selection in the same way^69^. Emotional distractors typically do not cause behavioural interference in healthy populations^54^. However, research shows robust neural emotional interference effects in healthy and clinical populations using the emotional counting Stroop task^51,59,60^; we therefore predicted that general negative and specific negative conditions would dissociate from the other task conditions to reveal a shared interference effect for negative compared to neutral words. In contrast to our predictions, the specific neutral words compared to specific negative words resulted in an aberrant interference effect that overlapped with regions of the right executive control network^70^, anterior and posterior cingulate and the left amygdala and hippocampus.

Positive emotional interference has been much less studied compared to negative emotional interference, however several studies have suggested that positive and negative distractors can result in similar behavioural interference if the stimuli are highly arousing^71,72^. We propose that our result is a product of stimulus selection. The focus of our participants’ stress was their occupation, thus it is possible that the teacher specific negative and neutral words were equally arousing to the study cohort. Interestingly, the pattern of activation identified for specific neutral words did not overlap with the previously reported emotional Stroop effects^59^, which could reflect an interaction between executive control, emotion, and memory that relates to the word type, frequency of use in day-to-day life, and associated symptoms of stress.

The focus of this study was on the interference effect as described previously^60^ and the difference between general and specific negative stimuli, we subsequently removed the specific neutral condition from the analysis. While we do not attempt to interpret the effect found for specific neutral words further, we do suggest future research is necessary to understand whether the neural activation is driven by valence or arousal of stimuli within the specific population being studied. It is important to highlight that accuracy on the specific neutral words was marginally reduced compared to all other conditions which suggests that neural interference, associated with counting neutral occupation relevant words, is associated with a quantifiable behavioural effect.

One advantage of the PLS analysis technique utilised in the current study is the ability to explore not only spatial but temporal patterns of neural activation. In the present study we found that early neural activation for negative words is related to greater activation of the OFC, precuneus, fusiform gyrus, as well as middle temporal regions and visual processing regions. This result partially supported our hypothesis that emotional interference during negative trials would result in increased activation of the amygdala, fusiform gyrus, DLPFC, insular cortex, ACC, and medial frontal regions^51^. The meta-analysis by Song and colleagues^51^ implicated the precuneus as having a role in intense emotional interference, however given a lack of behavioural evidence for this effect we suggest that that early neural activation of the OFC and fusiform may reflect strategic use of cognitive reappraisal^73^ to down regulate negative emotional experience. The lateral OFC has also been implicated in cognitive reappraisal strategies specifically for negative stimuli^74^. Amongst other strategies, Sutton and colleagues^75^ identified that teachers regularly utilise cognitive reappraisal to regulate emotions in a classroom setting. Critically, we did not find evidence of amygdala activation in response to negative stimuli. Activation of the amygdala has been reported in the generation of the emotional interference effect^57^ yet deactivation of the amygdala has been reported in the investigation of control over emotional interference^55^. Together with behavioural evidence, the results of this analysis suggest that teachers are regulating their emotional responses toward both general and occupation specific negative words.

A second, late peak of activation was identified through the spatiotemporal analysis of emotional interference which was found to differentiate teacher specific negative words from all other task conditions. Activation during general and control word trials reflected some commonality with the early pattern of activation for negative words but was much more distributed and included regions thought to be involved in counting, visual processing, and lexical processes^76,77^. In contrast, counting teacher-specific negative words was associated with reduced activation in these regions and increased activation of the left hippocampus. In isolation, activation of the hippocampus is difficult to interpret, however, given strong evidence suggesting that the hippocampus has a role in relational and associative processing^78^, one could tentatively speculate that processing occupation-specific negative words relies on additional associative processing demands. This argument aligns with literature suggesting stress improves or enhances memory for negative stimuli but impairs recall of neutral stimuli^79,80^. A critical future direction of this work is to explicitly ask participants to report on any emotion regulation strategies used during the task. Without the inclusion of a low/no stress group of teachers and a non-teacher control group, it is not possible to conclude that the observed effects are unique to a teacher cohort. However, these results provide a basis for future research in the field of occupational stress and emotional interference and indicate the importance of capturing the temporal dynamics of neural processing to fully understand these effects.

We address the question of whether individual variability in self-reported emotion dysregulation, cognitive control, and emotion recognition performance were related to neural activation during a task involving emotional interference. Interestingly, outcomes on each of these measures did not reveal any significant inter-correlations; and only the emotion dysregulation measure correlated with perceived stress. Our results suggest that in teacher’s suffering moderate to high stress, emotion recognition and cognitive control may not be directly associated with self-reported stress. However, individual variability in cognitive control and emotion recognition may still correlate with different patterns of neural activation during the emotional Stroop task.

The results of this study revealed, emotion dysregulation scores correlated with brain activations during the general *neutral* condition of the task. Greater emotion dysregulation was associated with increased activation of the precuneus, middle and lateral occipital regions and the fusiform gyrus; correlations were also found in the middle temporal cortices and inferior frontal gyrus. This result contrasts with our prediction that greater emotion dysregulation would be associated with greater engagement of regions involved in negative emotional interference. Rather, activations overlapped with regions commonly associated with cognitive control (i.e., the anterior insula), emotion processing and episodic memory (i.e., medial temporal lobes and angular gyrus). The control condition did not correlate with the pattern of activation suggesting that these data reflect a form of interference for neutral words that related to emotion regulation difficulties and stress.

Interestingly, the left anterior insular cortex has been suggested to have a role in cognitive inhibition, response inhibition and emotional interference^81^, and the insular cortex has been found to have greater activation in people who suffer anxiety suggesting that this region is hyper responsive to emotional valence^82^. One interpretation of our results is that people suffering from moderate to high stress, compounded by greater difficulties regulating their emotions, experience hyper reactivity toward ambiguously valenced stimuli. Hypersensitivity to neutral stimuli has been reported previously in other populations suffering emotion dysregulation. In one study it was found that patients with bipolar disorder (regardless of the current symptomatic status of their condition) showed heightened affective reactivity to neutral-valence scenes/images as well as decreased maintenance of affective response^83^. Individuals with a depression history (i.e., regardless of whether symptoms were in remission or current) have also been shown to have slower and less accurate recognition of emotionally neutral faces^84^. The results of Leppänen and colleagues^84^ suggest that depressed individuals perceive emotionality in faces depicting neutral expressions. A similar finding was reported by Svetieva and Frank^65^ who demonstrated that greater emotion dysregulation was associated with enhanced recognition of micro-expressions, in particular, anger. One limitation of the present study is that participants were not asked to rate valence or arousal of the stimuli which would have allowed us to explore this result in greater depth.

As expected, slow response times on the ERT were correlated with greater activation in regions associated with emotional processing, and visual word processing during the presentation of teacher-specific words. Slow response times were also associated with significantly reduced activation in these regions during the general negative and general neutral conditions of the task suggesting that teachers with poor emotion recognition show a unique neural differentiation for negative teacher-specific words. Significant activations were found in the left and right fusiform gyrus in regions associated with visual word processing^85^ and the fusiform face area (FFA).

The FFA does not only activate preferentially for faces, but has also been shown to preferentially respond to any over-trained stimulus or stimuli related to the subjects area of expertise^86^. Given that these activations increased only for teacher-specific negative words, it is possible that this activation reflects expertise with the stimuli. In support of this, slow response times on the ERT were associated with years of teaching after controlling for age. The ERT was also a facial recognition task, together this evidence could indicate that greater activation of the FFA for an area of expertise results in a trade-off that marginally impacts face processing. An important consideration in interpreting this result is the influence of age on neural processing which, in this study, ranged from 25 to 67 years. Emotion recognition was also found to correlate with years of teaching after controlling for the influence of age, but age and years of teaching are intrinsically related. Thus, it is possible that differences in the emotional Stroop task related to individual differences in emotion recognition could also reflect an underlying effect of age. In the future it would be interesting to separate positive and negative emotion recognition performance due to known selective deficits in the recognition of fear and anger in aging^87^.

In the present study we sought to explore individual differences in emotional interference among a group of moderate-high stress teachers. We anticipated stress and emotion dysregulation would relate to reduced inhibition and greater emotional interference for teacher specific negative words. The results of our group task-based analyses are in contrast to previous studies^51^. Due to limitations on the study design, we can only tentatively interpret these findings as evidence that despite high levels of stress, teachers may use cognitive reappraisal strategies to reduce the effect of emotional interference. Our results suggest that both negative general and specific words result in engagement of early cognitive control mechanisms. Patterns of neural activation temporally differentiate revealing sustained hippocampal activity for teacher-specific negative words, but not general negative words. These results suggest that control over the effect of emotional interference may be better understood through the use of neuroimaging techniques such as magnetoencephalography or electroencephalography that can more finely capture the temporal dynamics of neural processing. It would be particularly interesting to assess temporal differences in implicit and explicit emotion regulation responses in the context of individual variability and mental health outcomes.

In this study we identified a correlation between neural activation unique to the general neutral condition and emotion dysregulation. We argue that emotion dysregulation might relate to heightened emotional reactivity for neutral words, possibly due to their ambiguous nature, however future research should consider measuring arousal, and valence of the word stimuli in the sample population directly. Finally, we suggest that future research is needed to explore a potential trade-off between occupational expertise and emotion recognition due to joint reliance on the FFA, as this relates to aging. Despite the methodological challenges presented in this research, together, these findings give direction for future research in the field of emotional interference. These results also indicate that emotional interference in teachers suffering from moderate to high stress may be partly mediated by learned emotion regulation strategies and suggest that those suffering from greater difficulties in emotion regulation may show interference during neutral word processing.

## Methods

### Participants

Fifty-four participants were included in the present study. All participants were screened for magnetic resonance imaging (MRI)-compatibility (i.e., no metal implants) and had no history of neurological disease, trauma, or neuropsychological disorders (e.g., clinical depression/anxiety, epilepsy). Participants were right-handed, had no history of substance use, and did not take part in regular mindfulness-based practices (i.e., meditation/yoga) or vigorous exercise. The participants included in this study were initially recruited as part of a larger longitudinal stress-reduction intervention study which has been reported elsewhere^88^. In the present study, time one pre-intervention, data were analysed with the aim of exploring correlates between brain activity for emotion/cognitive control and self-reported stress, emotion recognition and cognitive flexibility. Participants were registered and practicing Australian (Queensland) teachers suffering from (self-reported/non-clinical) workplace stress and burnout. A total of 5 participants were excluded from the present research due to missing self-report data. Thus, the sample reported here consists of 49 participants (3 males, 46 females) ranging in age from 25 to 67 years of Age (*M*_age_ = 44.77 years; SD = 10.77 years). On average participants had 18.15 years of teaching experience (SD = 10.51), moderate-high perceived stress (*M* = 19.17*, SD* = 4.85) and a mean DERS score of 76.90 (*SD* = 18.23).

### Ethics

This study was approved by the University of Queensland Human Research Ethics Committee. Participation was voluntary and all participants provided informed written consent before participating in the study.

### Outcome measures

#### Self-report

The 36-item *Difficulties in Emotion Regulation Scale* (DERS^27^) was used to measure problems in participants’ emotion dysregulation. It yields a total score as well as scores on six subscales, including: Acceptance of emotions, Awareness of emotions, Clarity of emotions, Impulse control, Access to emotion regulation strategies, and Engagement in goal-directed behaviour. For this study, only the total DERS score was used. Responses are scored on a 5-point Likert scale (1 = Almost Never - 5 = Almost Always). The DERS total score ranges from 36 to 180, with higher scores indicating greater level of difficulty with emotion regulation.

Stress was measured using the *Perceived Stress Scale* (PSS^62^), a 10-item questionnaire assessing the degree to which situations in the respondent’s life are appraised as stressful. Responses are indicated on a 5-point scale ranging from 0 (never) to 4 (very often) and summed to form a total score, with higher scores representing greater levels of perceived stress.

#### CANTAB tasks

Participants completed a number of tasks from the Cambridge Neuropsychological Test Automated Battery^89^ administered using Apple iPads. Of relevance for the present study was the AST and the ERT.

*AST –* Participants have two buttons one on the left hand side of the screen and one on the right. Participants are first trained to respond to an arrow on the screen by pressing the button (left or right) that corresponds with the direction of the arrow. During the next training phase the arrow appears on either the right or left side of the screen and a cue is given at the top of the screen. The cue is either “SIDE” or “DIRECTION”. During SIDE cued trials, the participant is required to press the button that corresponds with the side of the screen that the arrow appears on, in the DIRECTION cued trials participants are asked to press the button that corresponds with the direction of the arrow regardless of the side of screen on which it is presented. In total, 160 experimental trails, with an equal number of side and direction cues, are presented in a randomised order.

*ERT –* Participants are shown 180 stimuli of faces across two blocks, each stimuli depicts one of 6 facial emotions. There are 15 different photographs of each of the emotions and these images depict different levels of intensity. The stimuli are derived from the facial features of real individuals but have been computer-morphed. Each face is displayed for 200 ms and then covered up, and the 6 button options (sadness, happiness, fear, anger, disgust and surprise) appear. Participants are asked to select the button which best describes the emotion of the face stimulus.

### fMRI task

#### Experimental task design

The emotional counting Stroop task, originally developed by Whalen and colleagues^60^ is an implicit emotion regulation variant of the counting Stroop task. The counting Stroop task was developed for use in function MRI experiments to minimise head motion by instructing participants to use a motor, instead of a spoken, response. In this study participants were asked to press a button to indicate the number of times (1–4) a word was presented on screen, regardless of the word meaning. The task involved four experimental conditions and a control condition. The control condition words were “one”, “two”, “three” and “four”. The number of times the word appeared on screen matched the word meaning (i.e., if the word was two, it appeared twice, and the participants responded with button 2). The experimental trials were separate into general words and teacher specific words, and the valence of these words was either neutral or negative. Words were presented on screen for 1000 ms and participants were instructed to respond as accurately as possible but to ignore the meaning of the word. Between each trial a jittered interstimulus interval of 1000–2000 ms was used where a fixation cross was presented. The task was separated into four blocks. The presentation of conditions was pseudorandomized within block and the presentation of blocks was counterbalanced across participants. Before the onset of each block, participants were reminded of the task instructions both auditorily and visually. The emotional counting Stroop task was presented using E-Prime (Version 2), standard edition. Responses were made on a 1 ×4 fibre optic response pad.

#### Task behavioural analysis

Performance on the emotional Stroop task was assessed through accuracy (%) and reaction time. Reaction times were adjusted for individual age-related differences by subtracting the participants average response time on the control trial from their average response time for each condition. Pearson’s correlations were calculated between the task conditions and numerous variables of interest including years of teaching experience, stress, and factors relating to individual emotion recognition. One way RM-ANOVA was used to explore differences among conditions on the emotional counting Stroop task in reaction time and accuracy.

#### Image acquisition and analysis

All images were acquired on a 3 Tesla Magnetom Trio Trim scanner with a 32-channel head coil. A T1-weighted volumetric anatomical MRI was acquired for each participant (MP2-RAGE). The following parameters were used: 176 slices sagittal; 1 mm^3^ isotropic volume; repetition time (TR) = 4000 ms; echo time = 2.89 ms; field of view = 256 mm. Functional MRIs were acquired using a T2*-weighted echo-planar image pulse sequence (45 slices, 2.5 mm slice thickness; voxel size 2.5 mm^3^, TR = 3000 ms; echo time = 30 ms; field of view = 190 mm; flip angle = 90 degrees). Four functional MRIs (corresponding to the four task blocks) were acquired, each with a length of 6 min and 6 s.

Brain activation was assessed using the blood oxygenation level-dependent effect^90^. Functional images were first preprocessed using Statistical Parametric Mapping software (SPM12; http://www.fil.ion.ucl.ac.uk/spm). All images were slice-time corrected, realigned to a mean image for head motion, spatially normalised to the Montreal Neurological Institute (MNI) template (voxel size 2 mm^3^), and spatially smoothed with a 6 mm full width maximum Gaussian kernel. Head motion did not exceed 2 mm in any of the data.

Whole-brain fMRI data was then analysed using an event-related PLS analysis. PLS is a model free multivariate tool which is statistically and conceptually similar to principal component analysis^91^. A key assumption of this analysis type is that cognitive processes are best analysed as the coordinated activity of groups of voxels rather than the independent activity of any single voxel^92,93^. In contrast to univariate fMRI analysis methods, multivariate methods can be utilised to identify groups of brain regions, distributed over the entire brain, whose activity changes as a function of task demands or as a function of behavioural performance.

PLS mean-centres and then decomposes the covariance matrix between brain activity and the experimental design (or an external variable such behaviour or age) for all participants in a single analytic step. This is achieved using SVD which generates a weight for each voxel, and designates its degree of covariance with the whole brain activity pattern. As PLS is a multivariate technique, no apriori constraints are placed on the data, rather each analysis was exploratory in nature. SVD maximally accounts for covariance in the data and results in separate, mutually orthogonal LVs, which describe patterns of brain activity related to the experimental design^91,92^. The percentage of covariance that is calculated for each LV reveals its relative importance. PLS also outputs a spatiotemporal map of saliences indicating the degree to which each voxel relates to the LV. Additionally, brain scores are calculated in PLS for each participant and indicate how strongly the whole-brain pattern of voxel saliences is related to each experimental condition, in each LV. Furthermore, a condition of PLS is that no two LVs are correlated with one another^92^. PLS then assesses the statistical significance of each LV using permutation testing with 500 permutations^94^. Reliability of the pattern of brain activity patterns for each voxel is assessed using a bootstrapping procedure with 100 bootstraps. This procedure results in an estimate of the standard error which is used to calculate the bootstrap ratio^95^. Peak voxels with a minimum bootstrap ratio of 3 are considered to be reliable^96^. In PLS, computation of LVs and corresponding brain images is conducted in a single analytic step across all voxels and participants; therefore, no correction for multiple comparisons is required^93^. Finally, a brain score, indicating how strongly each resulting pattern is expressed in each individual participant, is calculated by multiplying each individual data set with the whole-brain activation loadings. In this study we report peak clusters of greater than 20 voxels, all results figures are reported in MNI coordinate space using the Harvard Oxford Atlas.

### Reporting summary

Further information on experimental design is available in the Nature Research Reporting Summary linked to this paper.

## Supplementary information

Supplementary Information

Reporting Summary Checklist

## Data Availability

The data that support the findings of this study are available from the corresponding author upon reasonable request.
