# Supplementary material for: Neural activation during emotional interference corresponds to emotion dysregulation in stressed teachers
Source: NPJ Sci Learn. 2022 Apr 20;7:5. doi: 10.1038/s41539-022-00123-0 (PMC9021303; doi:10.1038/s41539-022-00123-0)
Supplement: Supplementary file 1 — Supplementary Information [file 41539_2022_123_MOESM1_ESM.pdf]

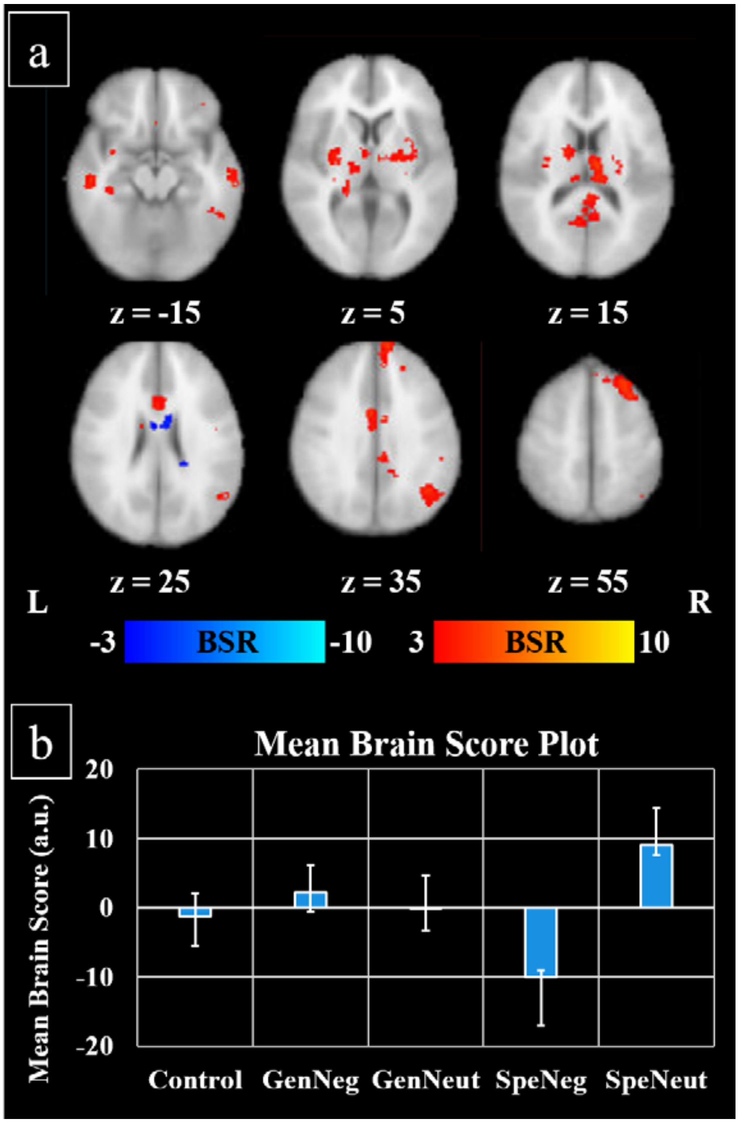

Supplementary Figure 1.

Whole-brain PLS including all emotional Stroop task conditions (control, general negative, general neutral, specific negative, and specific neutral). **(a)** Latent variable voxel saliences overlaid on the 2mm MNI-average brain template differentiated activation during the specific neutral condition (red) from an anticorrelated pattern of activation engaged during the specific negative condition (blue). Red and blue patterns of activation were anticorrelated meaning that greater activation in red regions relates to decreased activation in blue regions. BSR = bootstrap ratio tests of significance; L = left hemisphere; R = right hemisphere; z = axial slice number. **(b)** Mean brain scores (a.u. = arbitrary units) indicating the mean contribution of each task condition to the activation patterns displayed in (a). Error bars reflect 95% confidence intervals from the bootstrap analysis.

Supplementary Table 1.

Peak coordinates for significant activations differentiating the specific neutral and specific negative words.

All Condition LV1 (Lag 1)							
**Specific Neutral (Positive Saliences)**							
		**MNI Coordinates**					
**Region**	**Hem**	**x**	**y**	**z**	**BSR**	**Cluster Size**	**p val**
Frontal Pole	R	8	56	32	5.36	225	< .001
MFG	R	28	22	56	5.33	447	< .001
MTG (posterior division)	L	-52	-20	-16	4.94	63	< .001
	R	62	-24	-16	4.23	68	< .001
ITG (temporooccipital part)	R	52	-50	-18	3.72	79	< .001
MTG (temporooccipital part)	L	-56	-44	-10	3.9	36	< .001
Temporal Pole	R	62	4	-20	3.82	25	< .001
Precuneus	L	-8	-60	14	3.76	32	< .001
	R	8	-54	18	3.87	73	< .001
SMG (anterior division)	R	52	-34	36	3.92	22	< .001
Lateral Occipital Cortex (superior division)	R	52	-64	30	4.76	439	< .001
CG (anterior division)	L	-6	4	34	4.88	269	< .001
CG (posterior division)	R	2	-40	18	5.24	106	< .001
Subcallosal Cortex	R	6	24	-14	3.72	43	< .001
Thalamus	L	-8	-4	-2	3.66	27	< .001
	R	8	-12	12	5.01	217	< .001
Putamen	L	-32	-6	8	5.14	427	< .001
	R	22	-10	10	4.34	256	< .001
Hippocampus	L	-20	-38	4	4.24	62	< .001
Amygdala	L	-36	0	-18	3.94	21	< .001
Brain Stem	L	-4	-52	-38	4.14	30	< .001
**Specific Negative (Negative Saliences)**							
Caudate (Body)	R	8	0	22	-4.46	39	< .001

Notes: MFG = middle frontal gyrus; MTG = middle temporal gyrus; ITG = inferior temporal gyrus; SMG = supramarginal gyrus; CG = cingulate gyrus; BSR = bootstrap ratio from the PLS analysis; Hem = hemisphere; R = right; L = left; p val = estimated p value from the BSR. All results were taken from lag one of the spatiotemporal analysis corresponding to 3-6 seconds after stimulus onset. Nomenclature correspond to the Harvard-Oxford cortical and subcortical structural atlases.

Supplementary Table 2.

Regions engaged during the emotional interference task that are significantly correlated with emotion recognition response latencies.

Significant correlation with ERT: LV1 (Lag 4)							
		MNI Coordinates					
Region	Hem	x	y	z	BSR	Cluster Size	p val
Lateral Occipital Cortex, inferior division	R	36	-70	2	7.11	2512	< .001
Precuneus	L	-32	-56	10	6.49	3161	< .001
SPL	R	26	-46	48	3.99	72	< .001
Supramarginal Gyrus, anterior division	R	6.56 8	-32	26	5.04	228	< .001
Supramarginal Gyrus, posterior division	L	-52	-36	34	4.08	44	< .001
	R	46	-46	28	4.02	25	< .001
STG, posterior division	L	-52	-20	-2	5.72	231	< .001
MTG, anterior division	R	50	-6	-26	4.8	122	< .001
MTG, posterior division	R	42	-40	-6	6.19	186	< .001
Postcentral Gyrus	L	-26	-36	56	5.01	470	< .001
	R	34	-26	50	5	287	< .001
Precentral Gyrus	L	-40	-10	28	5.24	1220	< .001
	R	62	12	16	4.81	117	< .001
Frontal Pole	L	-34	56	10	3.79	33	< .001
	R	20	54	6	4.67	152	< .001
SFG	L	-24	-2	70	4.67	30	< .001
	R	16	16	50	4.43	72	< .001
MFG	R	32	0	66	4.36	72	< .001
IFG, pars triangularis	R	38	34	8	3.61	20	< .001
OFC	L	-36	32	-8	3.9	26	< .001
	R	38	24	-12	3.57	22	< .001
Frontal Operculum Cortex	L	-42	16	6	4.14	287	< .001
Central Opercular Cortex	R	48	4	-2	4.72	273	< .001
Caudate (Body)	R	24	-10	28	5.49	129	< .001
Caudate (Head)	R	14	22	-8	3.94	24	< .001
Paracingulate Gyrus	L	-18	50	8	3.86	26	< .001
	R	16	32	30	4.28	25	< .001
CG, anterior division	L	-16	30	12	5.41	76	< .001
	R	6	-8	34	4.19	208	< .001
CG, posterior division	L	-6	-18	30	4.44	35	< .001
	R	16	-44	30	4.78	103	< .001

Thalamus	L	-8	-20	4	4.83	373	< .001
Putamen	L	-34	-32	2	5.61	960	< .001
Pallidum	R	18	-8	-2	4.58	64	< .001
Cerebellum	L	-30	-86	-24	5.2	157	< .001
	R	24	-42	-30	4.5	25	< .001

Notes: SPL = superior parietal lobule; STG = superior temporal gyrus; MTG = middle temporal gyrus; SFG = superior frontal gyrus; MFG = middle frontal gyrus; IFG = inferior frontal gyrus; OFC = orbitofrontal gyrus; CG = cingulate gyrus; BSR = bootstrap ratio from the PLS analysis; Hem = hemisphere; R = right; L = left; p val = estimated p value from the BSR. All results were taken from lag four of the spatiotemporal analysis corresponding to 12-15 seconds after stimulus onset. Nomenclature correspond to the Harvard-Oxford cortical and subcortical structural atlases.
