# Supplementary material for: Neural activation during emotional interference corresponds to emotion dysregulation in stressed teachers
Source: NPJ Sci Learn. 2022 Apr 20;7:5. doi: 10.1038/s41539-022-00123-0 (PMC9021303; doi:10.1038/s41539-022-00123-0)
Supplement: Supplementary file 2 — Reporting Summary Checklist [file 41539_2022_123_MOESM2_ESM.pdf]

nature portfolio

Corresponding author(s): Dr. Samuel Fynes-Clinton

Last updated by author(s): Mar 10, 2022

# Reporting Summary

Nature Portfolio wishes to improve the reproducibility of the work that we publish. This form provides structure for consistency and transparency in reporting. For further information on Nature Portfolio policies, see our Editorial Policies and the Editorial Policy Checklist.

## Statistics

For all statistical analyses, confirm that the following items are present in the figure legend, table legend, main text, or Methods section.

| n/a | Confirmed | |
|---|---|---|
| ☐ | ☒ | The exact sample size (*n*) for each experimental group/condition, given as a discrete number and unit of measurement |
| ☐ | ☒ | A statement on whether measurements were taken from distinct samples or whether the same sample was measured repeatedly |
| ☐ | ☒ | The statistical test(s) used AND whether they are one- or two-sided<br>*Only common tests should be described solely by name; describe more complex techniques in the Methods section.* |
| ☐ | ☒ | A description of all covariates tested |
| ☐ | ☒ | A description of any assumptions or corrections, such as tests of normality and adjustment for multiple comparisons |
| ☐ | ☒ | A full description of the statistical parameters including central tendency (e.g. means) or other basic estimates (e.g. regression coefficient) AND variation (e.g. standard deviation) or associated estimates of uncertainty (e.g. confidence intervals) |
| ☐ | ☒ | For null hypothesis testing, the test statistic (e.g. *F*, *t*, *r*) with confidence intervals, effect sizes, degrees of freedom and *P* value noted<br>*Give P values as exact values whenever suitable.* |
| ☒ | ☐ | For Bayesian analysis, information on the choice of priors and Markov chain Monte Carlo settings |
| ☐ | ☒ | For hierarchical and complex designs, identification of the appropriate level for tests and full reporting of outcomes |
| ☐ | ☒ | Estimates of effect sizes (e.g. Cohen's *d*, Pearson's *r*), indicating how they were calculated |

*Our web collection on statistics for biologists contains articles on many of the points above.*

## Software and code

Policy information about availability of computer code

| | |
|---|---|
| Data collection | The emotional counting Stroop task was presented using E-Prime (Version 2), standard edition. Responses were made on a 1 x 4 fibre optic response pad. The Cambridge Neuropsychological Test Automated Battery (CANTAB, 1999) was administered using Apple iPads. |
| Data analysis | Functional images were first preprocessed using Statistical Parametric Mapping software (SPM12; http://www.fil.ion.ucl.ac.uk/spm). All fMRI data was then analysed using event-related Partial Least Squares analysis (PLS; https://www.rotman-baycrest.on.ca/index.php?section=84). |

For manuscripts utilizing custom algorithms or software that are central to the research but not yet described in published literature, software must be made available to editors and reviewers. We strongly encourage code deposition in a community repository (e.g. GitHub). See the Nature Portfolio guidelines for submitting code & software for further information.

## Data

Policy information about availability of data

All manuscripts must include a data availability statement. This statement should provide the following information, where applicable:

- Accession codes, unique identifiers, or web links for publicly available datasets
- A description of any restrictions on data availability
- For clinical datasets or third party data, please ensure that the statement adheres to our policy

The data that support the findings of this study are available from the corresponding author upon reasonable request.

# Field-specific reporting

Please select the one below that is the best fit for your research. If you are not sure, read the appropriate sections before making your selection.

☐ Life sciences ☒ Behavioural & social sciences ☐ Ecological, evolutionary & environmental sciences

For a reference copy of the document with all sections, see nature.com/documents/nr-reporting-summary-flat.pdf

# Behavioural & social sciences study design

All studies must disclose on these points even when the disclosure is negative.

| | |
|---|---|
| Study description | Neuroimaging study of practicing teachers suffering from workplace stress and burnout to examine individual differences and neural correlates of emotional interference. |
| Research sample | Fifty-four registered and practicing Australian (QLD) teachers suffering from (self-reported/non-clinical) workplace stress and burnout were recruited. The sample reported here consists of 49 participants (3 males, 46 females) ranging in age from 25-67 years of age (mean age = 44.77 years; SD = 10.77 years). On average participants had 18.15 years of teaching experience (SD = 10.51), moderate-high perceived stress (M = 19.17, SD = 4.85) and a mean DERS score of 76.90 (SD = 18.23). |
| Sampling strategy | A priori sample size calculations were not performed. Recruiting registered and practicing teachers was challenging because testing needed to occur outside of the classroom hours. Our approach was simply to recruit as many participants as possible.<br><br>To assess statistical power a G*power (version 3.1.9.4; Faul et al., 2007) analysis using the repeated measures ANOVA protocol was conducted. In order to detect a medium effect size (f = .25) for the analysis of fMRI task performance, with 80% power at an alpha .05 threshold, 21 participants would be required.<br><br>The partial least squares method used to analyse fMRI data in this study is a non-parametric and multivariate statistical method. These methods are particularly advantageous for examining smaller sample sizes and bootstrap resampling is recommended for small and/or non-homogeneous samples (Adèr et al., 2008). |
| Data collection | Participants were examined by a radiographer for MRI safety prior to completing the MRI task. Once in the MRI scanner, participants were presented with the fMRI task, and asked to respond using to the task using a 1x4 fibre optic response pad. After the MRI scan, participants completed a pen and paper questionnaire containing the self-report measures described in this study and complete the CANTAB neuropsychological tests on an Apple iPad. |
| Timing | The data used in this study were collected across a four month period between 30 Mar 2016 and 19 July 2016. |
| Data exclusions | A total of 5 participants were excluded from the present research due to missing self-report data. |
| Non-participation | No participants dropped out of the study. |
| Randomization | The participants of this study were recruited for a larger longitudinal stress-reduction intervention study. The data and results described in this study involve only the pre-intervention data, prior to randomization into intervention groups. |

# Reporting for specific materials, systems and methods

We require information from authors about some types of materials, experimental systems and methods used in many studies. Here, indicate whether each material, system or method listed is relevant to your study. If you are not sure if a list item applies to your research, read the appropriate section before selecting a response.

## Materials & experimental systems

| n/a | Involved in the study | |
|---|---|---|
| ☒ | ☐ | Antibodies |
| ☒ | ☐ | Eukaryotic cell lines |
| ☒ | ☐ | Palaeontology and archaeology |
| ☒ | ☐ | Animals and other organisms |
| ☐ | ☒ | Human research participants |
| ☒ | ☐ | Clinical data |
| ☒ | ☐ | Dual use research of concern |

## Methods

| n/a | Involved in the study | |
|---|---|---|
| ☒ | ☐ | ChIP-seq |
| ☒ | ☐ | Flow cytometry |
| ☐ | ☒ | MRI-based neuroimaging |

# Human research participants

Policy information about studies involving human research participants

| | |
|---|---|
| Population characteristics | see above |
| Recruitment | Participants were registered and practicing Australian (QLD) teachers suffering from (self-reported/non-clinical) workplace stress and burnout. Participants were right-handed, had no history of substance use, and did not take part in regular mindfulness-based practices or vigorous exercise. All participants were screened for MRI-compatibility, had no history of neurological disease, trauma, or neuropsychological disorders. The participants included in this study were initially recruited as part of a larger longitudinal stress-reduction intervention study which has been reported elsewhere (Carroll et al., 2021a). |
| Ethics oversight | This study was approved by the University of Queensland Human Research Ethics Committee. Participation was voluntary and all participants provided informed written consent before participating in the study. |

Note that full information on the approval of the study protocol must also be provided in the manuscript.

# Magnetic resonance imaging

## Experimental design

| | |
|---|---|
| Design type | Event related |
| Design specifications | The task was presented across 4 blocks corresponding to the 4 functional MRI scanning runs), each block contained, 75 trials (15 per condition). The presentation of conditions was pseudorandomized within block and the presentation of blocks was counterbalanced across participants. |
| Behavioral performance measures | Button press accuracy and response times were recorded. All subjects performed at greater than 85% accuracy indicating that they understood and complied with the task demands. |

## Acquisition

| | |
|---|---|
| Imaging type(s) | Functional and structural |
| Field strength | 3 Tesla |
| Sequence & imaging parameters | A T1-weighted volumetric anatomical MRI was acquired for each participant (MP2-RAGE). The following parameters were used: 176 slices sagittal; 1 mm3 isotropic volume; repetition time (TR) = 4000 ms; echo time (TE) = 2.89 ms; FOV = 256 mm. Functional MRIs were acquired using a T2*-weighted echo-planar image pulse sequence (45 slices, 2.5 mm slice thickness; voxel size 2.5 mm3, TR = 3000 ms; TE = 30 ms; FOV = 190 mm; flip angle = 90 degrees). Four functional MRIs (corresponding to the four task blocks) were acquired, each with a length of 6min and 6 sec. |
| Area of acquisition | Whole brain scan |

Diffusion MRI ☐ Used ☒ Not used

## Preprocessing

| | |
|---|---|
| Preprocessing software | All images were slice-time corrected, realigned to a mean image for head motion, coregistered to the subjects structural image, segmented, normalized, and spatially smoothed with a 6 mm full width maximum Gaussian kernel using SPM12. |
| Normalization | The in-built normalization procedures of SPM12 were used, including affine regularization to the MNI152 linear template. 4th degree B-spline interpolation was used. |
| Normalization template | The default template of SPM12, and thus used in the present study is the MNI152 template. |
| Noise and artifact removal | none |
| Volume censoring | none |

## Statistical modeling & inference

| | |
|---|---|
| Model type and settings | Partial least squares (whole-brain task & brain-behaviour) correlation, and mass univariate (behavioural data only). |
| Effect(s) tested | No a-priori constraints were placed on the analyses of MRI data rather, PLS is a data driven approach conceptually similar to principal component analysis whereby mutually orthogonal latent variables that best describe the covariance in the data are extracted. All behavioral data were analyzed using a one-way Analysis of Variance (two-sided). |

Specify type of analysis: ☒ Whole brain ☐ ROI-based ☐ Both

Statistic type for inference
(See Eklund et al. 2016)

PLS mean-centers and then decomposes the covariance matrix between brain activity and the experimental design (or an external variable such behavior or age) for all participants in a single analytic step using singular value decomposition. Statistical inference is implemented using bootstrapping procedures. Peak voxels with a minimum bootstrap ratio of 3 are considered to be reliable.

Correction

In PLS, latent variables are computed in a single analytic step across all voxels and participants using singular value decomposition; therefore, no correction for multiple comparisons is required (McIntosh & Lobaugh, 2004).

## Models & analysis

| n/a | Involved in the study |
| --- | --- |
| ☒ | ☐ Functional and/or effective connectivity |
| ☒ | ☐ Graph analysis |
| ☐ | ☒ Multivariate modeling or predictive analysis |

Multivariate modeling and predictive analysis

Whole-brain fMRI data was then analysed using an event-related Partial Least Squares analysis (PLS; https://www.rotman-baycrest.on.ca/index.php?section=84) which is conceptually very similar to principal components analysis. The data is mean-centered and then the covariance matrix between brain activity and the experimental design is decomposed using singular value decomposition. This procedures generates a weight for each voxel indicating its degree of covariance with the whole brain activity pattern. Output from the PLS analysis are mutually orthogonal latent variables describing patterns of activity related to the experimental design or external variables of interest (i.e. emotion dysregulation scores).
